# Retail food environment assessments: a case study of consumer and community nutrition environment perspectives of Logan, Utah

**DOI:** 10.1186/s12889-026-27398-x

**Published:** 2026-05-16

**Authors:** Abiodun T. Atoloye, Oluyemisi Akinsola, Habiba Nur, Heidi LeBlanc, Lea Palmer, Cris Meier

**Affiliations:** 1 Department of Nutrition, Dietetics, and Food Sciences, Utah State University, 8700 Old Main Hill, Utah, 84322-8700 USA; 2 Utah State University, Extension Home and Community, Utah, USA; 3 Create Better Health Hub, Utah State University, Utah, USA; 4 Department of Sociology, Social Work, and Anthropology & Extension, Utah State University, Utah, USA

**Keywords:** Community Nutrition Environment, Consumer Nutrition Environment, Community Nutrition Environment Score, Food retail outlets

## Abstract

**Background:**

Retail food environments are unevenly distributed in communities, often leading to structural inequities. Understanding these inequities is critical for designing effective, equity-focused interventions, especially in understudied smaller metropolitan areas. The consumer nutrition environment, which include in-store factors and community nutrition environments, covering neighborhood-level access both influence food purchasing behaviors. Measuring these environments provides a comprehensive view of factors shaping food choices, essential for targeted improvements in healthy food access and equity. This study examined the healthiness of food available in Logan, a growing small metropolitan area with a distinctive “main street food corridor”.

**Methods:**

A comprehensive food store audit was conducted using existing validated food audit tool across various store types and categories. Kernel density analysis in ArcGIS Pro was used to assess the location, type, and healthfulness of different food retail outlets throughout Logan based on Consumer Nutrition Environment Scores (CNES).

**Results:**

Key findings show that Logan’s foodscape is dominated by fast-food and full-service restaurants, making up over half of all stores. Our study revealed significant variability in CNES across different store types and categories with supermarkets and healthy specialty stores scoring highest, an indication of a better consumer nutrition environment, while convenience stores with gas stations scoring lowest, reflecting poorer support for healthy choices. Healthy specialty stores had superior CNES at the store-category level.

**Conclusion:**

While disparities exist in the availability of healthy food options across store types in Logan, the dominance of fast-food and full-service restaurants presents both challenges and opportunities to promote healthier options through partnerships and interventions.

**Supplementary Information:**

The online version contains supplementary material available at 10.1186/s12889-026-27398-x.

## Introduction

In the United States, a substantial share of food expenditures (ranging from 3% to 58%) occurs within various retail food outlets, including grocery stores, supermarkets [1, 2]. These outlets collectively known as retail food environment, is a physical and economic environment where food is sold. It encompasses the types, locations, and quality of food outlets available such as grocery stores, supermarkets, farmers markets, convenience stores, restaurants, and fast-food outlets. Importantly, it plays a key role in shaping food access, purchasing behaviors, and dietary habits of local residents [3, 4].

In addition to the retail food environment, two other important contexts influence food access and purchasing behaviors: the consumer nutrition environment and the community nutrition environment [5, 6]. The consumer nutrition environment refers to the in-store factors that influence food purchasing decisions, including food availability, pricing, promotional strategies, placement of products, and access to nutrition information [5, 6]. For example, whether fresh produce is placed at eye level, or whether sugary snacks are promoted at checkout counters can significantly affect consumer choices. The community nutrition environment, on the other hand, encompasses broader neighborhood-level factors that shape an individual’s ability to access food outlets in the first place [5, 6]. These include the geographic distribution of food retailers, transportation availability, neighborhood safety, and socioeconomic conditions. In essence, the community nutrition environment determines who can physically and economically access the food retail environment, and to what extent.

The retail food environment in urban areas is not always equally distributed. Previous studies have identified two structural inequities within the food retail environment: food deserts and food swamps [7, 8]. Food deserts occur in low-income and under-resourced areas, often geographically defined by census tracts or neighborhood block groups, where residents have limited access to full-service grocery stores or supermarkets that sell fresh, healthy food [9, 10]. These neighborhoods typically lack affordable options for fruits, vegetables, and whole grains. Food swamps, on the other hand, refer to geographic areas that have access to an abundance of food sources, however, there is an abundance of unhealthy food retailers (e.g., fast food restaurants, convenience stores) and characterized by high exposure to unhealthy food marketing [11]. While both concepts laid important foundation for food environment research by shifting attention from individual dietary choices to the structural and spatial factors that influence food access and diet. However, more recent research has highlighted limitations in these concepts and the need for more comprehensive approaches [12, 13]. For example, while food deserts measurement relies on barriers to healthy food access (such as transport, proximity, and poverty level), living in a food desert itself may not strongly influence people’s perceived food access or eating habits [14, 15]. Instead, how individual navigate and use the nearby food options matters more. Furthermore, in urban food deserts, proximity to supermarkets alone has not consistently been shown to improve dietary quality [16]. Other factors including food affordability, individual preferences, and cultural relevance which play critical role in shaping food purchasing behaviors [5] are not captured when relying on geographic proximity and barrier presented in food desert measurement.

Food swamp measurement typically overemphasizes the physical presence of food outlets, and the prevalence of unhealthy food options overlooking other critical factors such as food affordability, and socioeconomic, and cultural dynamics that influence food purchasing and consumption patterns. In many studies, food retailers are broadly categorized as “healthy” (e.g., supermarkets and farmers’ markets) or “unhealthy” (e.g., fast-food restaurants and convenience stores) based primarily on store type. However, growing evidence suggests that these binary classifications may oversimplify the complexity of food retail environments and overlook important in-store characteristics such as the availability, price, and quality of healthier food options [5]. Recent research has therefore called for more multidimensional approaches that capture both accessibility and the relative availability of healthy foods within stores rather than relying solely on outlet density or store typology [12]. These limitations highlight the importance of assessing consumer nutrition environments using composite measures that consider multiple in-store characteristics that shape food purchasing behaviors. A broader and holistic understanding of community food access with a focus on these critical factors is essential for designing effective, equity-focused interventions that enhance access to healthy and affordable food. Additionally, most urban studies focus on large metropolitan areas [7, 17, 18], which make it difficult to generalizations findings to smaller metropolitan areas. Examining the local food environment in small metropolitan areas will allow for a better understanding about the applicability of the assessment tools used in larger urban areas.

### Contextual background of Logan, Utah

Logan is a small metropolitan city experiencing a fast growing racial and ethnically diverse population, which is situated in Northen Utah. The area is comprised of 17 resident-populated census tracts encompassing a total population of 53,923 as of 2023, with racial and ethnic composition that includes about 74.2% White non-Hispanic, 15.4% Hispanic or Latino of any race, 3.3% Asian, 2.0% Black, 0.5% Native Hawaiian/Pacific Islander, and 10.3% identifying as two or more races [19]. The city’s median household income is approximately $60,687, and about 22.3% of residents live below the poverty line [19].

**Fig. 1:**
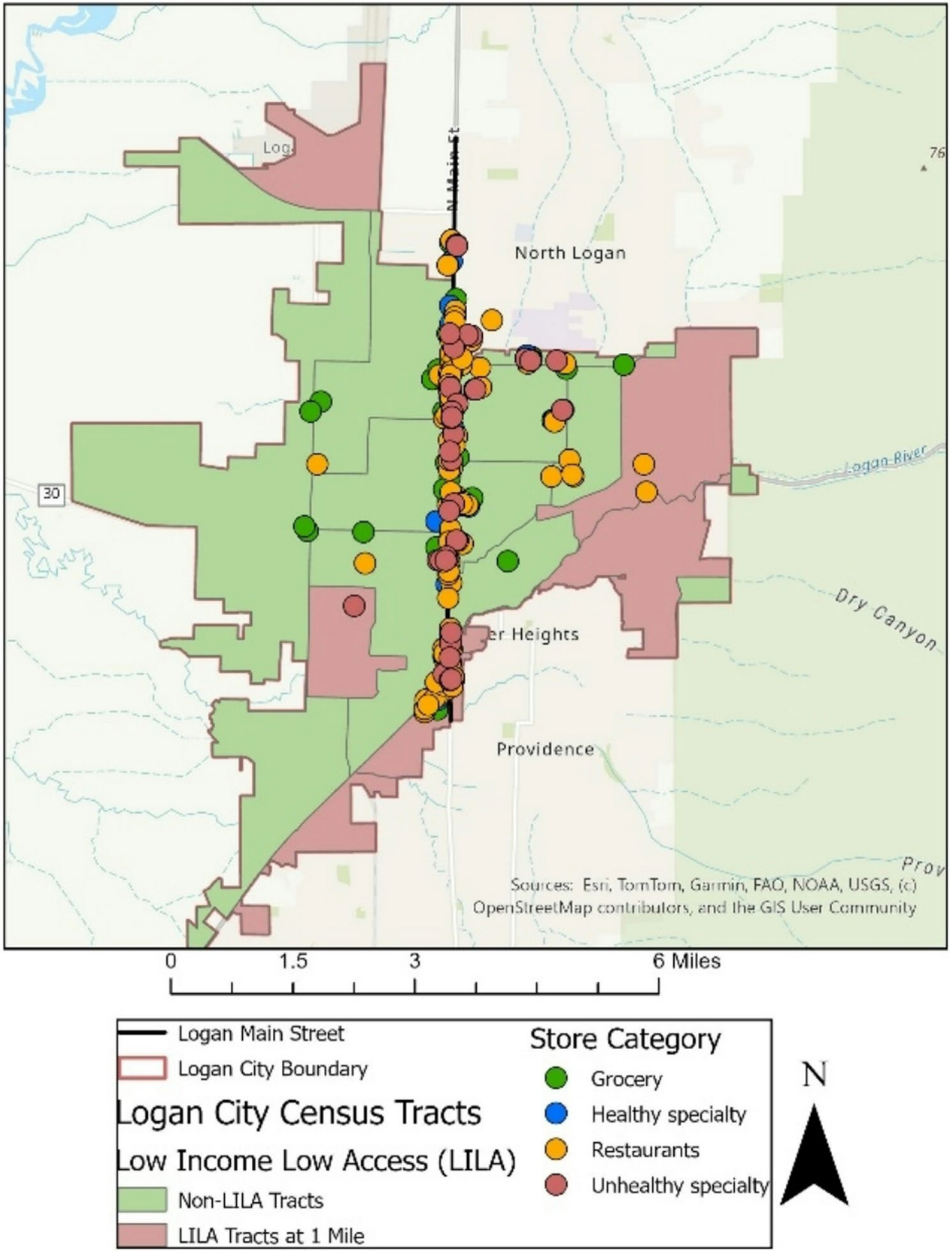
Low-Income, Low-Access (LILA) tracts at the 1-mile threshold and the distribution of different food stores in Logan, Utah

In recent years, Logan, Utah, has experienced notable changes in its foodscape that matches the growing population in the area [20], reflecting broader trends seen in other rapidly growing cities [21, 22]. This may be due to urbanization, population growth, changing retail patterns, and evolving consumer behaviors. Logan’s foodscape is best characterized as a “main street food corridor”, or “corridor-based food environment” marked by a linear distribution of food retailers concentrated along a central, high-traffic area that functions as both a commercial and social hub. This type of main street food corridor, while untypical compared to more evenly distributed foodscapes as it presents several strengths and community assets. The concentration of grocery stores, restaurants, and food vendors in one area enhances accessibility for those living nearby. Moreover, the Cache Valley Transit District (CVTD) offer free transit service that helps connect residents in outlying neighborhoods to retail food outlets in Logan. However, depending on the quality and type of available food, some areas may still experience localized food deserts or swamps. For example, according to 2019 data from the Food Access Research Atlas [23], six of the 17 census tracts were identified as Low-Income, Low-Access (LILA) areas at the 1-mile threshold (Fig. 1).

Understanding not only the types and locations of food stores but also the extent to which this distinctive “main street food corridor” supports healthy eating is essential for identifying the unique needs and challenges of this understudied metropolitan foodscape. Assessing how such foodscapes influence food access is also crucial for understanding the impact of urban planning decisions on local food environments. This study will use a food store audit to assess in-store offerings and the spatial distribution of food outlets, providing insights into the city’s overall food environment healthiness.

## Methodology

### Study design and setting

This single-case observational study assessed the food retail environment in Logan, Utah. An initial comprehensive list of food retail locations in the city (*N* = 659) was compiled from a purchased license retail businesses list from Utah Department of Commerce [24] to extract businesses in Logan (*n* = 484), online Yellow Pages (*n* = 12), Google Maps (*n* = 156), Yelp, and Facebook comments (*n* = 7). Using North America Industry Classification System (NAICS) codes [25] (a standardized federal classification system that categorizes businesses based on primary economic activity), businesses were excluded from the licensed retail businesses list if their NAICS codes did not correspond to food retail or food service categories. The duplicates among the data sources were eliminated (*n* = 226). Store addresses were geocoded using the US Census Geocoder [26], and the resulting latitude and longitude coordinates were imported into ArcGIS Pro [27] to filter out any stores located outside Logan city limits (*n* = 80).

**Fig. 2:**
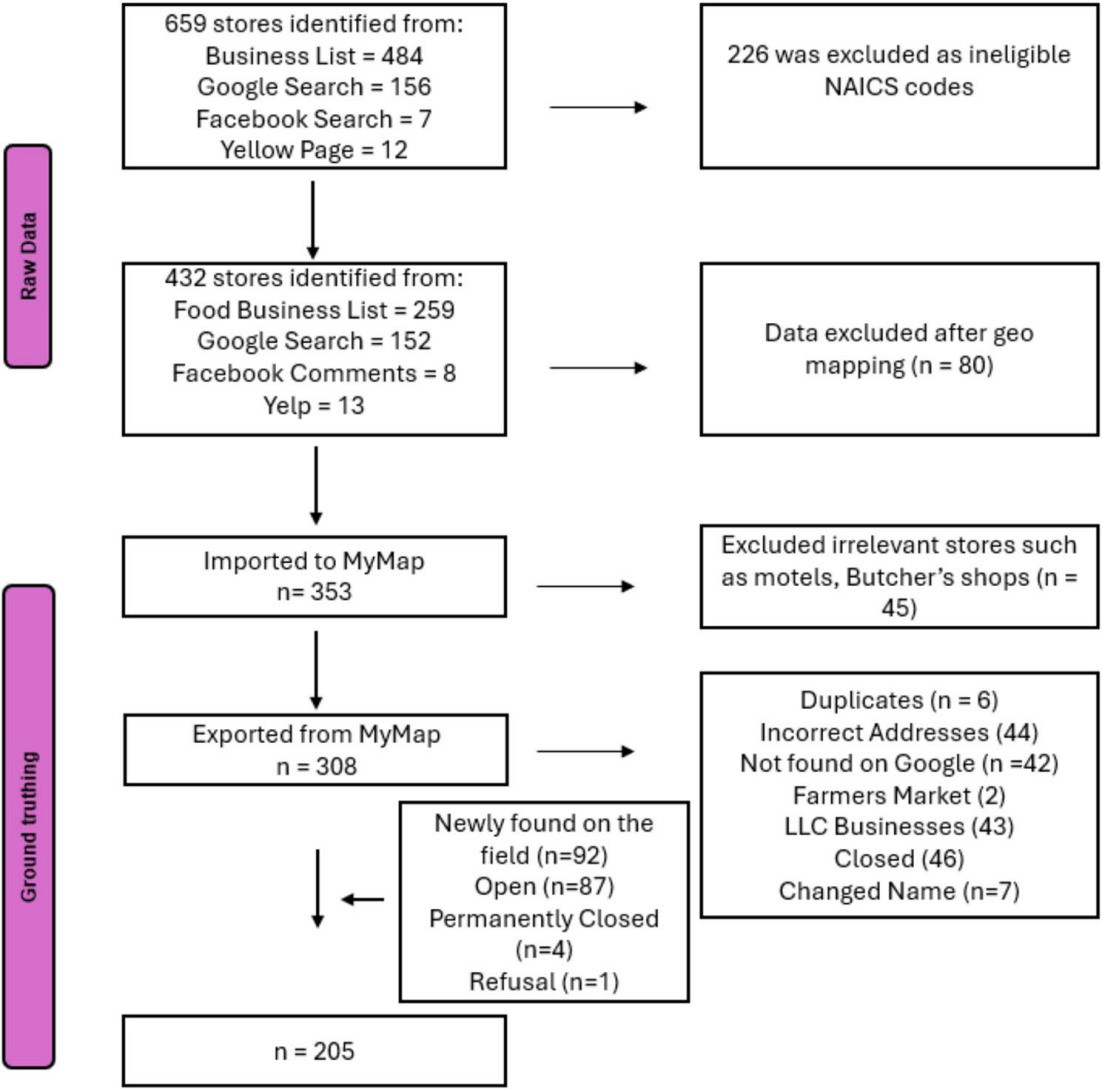
Flow Chart Detailing Food Store Selection from Mixed Sources

Next, additional data cleaning was needed to remove reoccurring duplicates (*n* = 6), incorrect addresses (*n* = 44), farmers’ markets (*n* = 2), permanently closed stores (*n* = 46), could not be found on Google search (*n* = 42), non-food businesses (*n* = 43), and change of business name (*n* = 7) via online information search. Additionally, farmers’ markets were excluded because no validated audit tool exists for this type of food retailer, resulting in a sample of 118 food outlets. Static maps displaying the filtered store data points were created in MyMap [28] to serve as guides for auditors during ground truthing and store audit visits. However, during the ground truthing and audits, 87 new food stores were identified and added. Figure 2 describes the process in detail.

### Store classification

Appendix A includes the definitions used to identify the different types of stores included in the study. These store types were further grouped into four categories for additional analytical purposes. These definitions were adapted from established sources [25].

### Food store audit tool

The study’s food store audit tool was adapted from existing validated audit tools, including the Nutrition Environment Measures Survey (NEMS) [29] and the Market Basket Assessment Tool (MBAT) [30] for the data collection. Two experts in nutrition and food access provided feedback before the final draft was programmed in REDCap [31]. The tool included five core domains: store identification; accessibility and accommodation; infrastructure and equipment; product placement and promotion; menu offerings and availability, price, quality, and variety of major food groups (grains, fruits, vegetables, meat/fish products, dairy, and legumes). Freshness was included for fruits, vegetables, meat/fish products. Others include sweetened beverages and snacks. Additional components covered the availability of healthy options, the presence of facilitators, and barriers to healthy options for restaurants and specialty stores. In this study, “healthy” foods were defined in alignment with the Dietary Guidelines for Americans [32], emphasizing nutrient-dense food groups such as fruits, vegetables, whole grains, legumes, lean proteins, and dairy products [29]. Rather than categorizing retailers as inherently “healthy” or “unhealthy,” this study evaluated the extent to which stores supported access to nutrient-dense foods through in-store characteristics such as availability, variety, freshness, and affordability using the validated framework established by NEMS [29, 30, 33, 34].

### Data collection procedures

Two trained research assistants conducted in-person pretest of the audit tool in two stores using the mobile version of REDCap to evaluate its usability and functionality in the field. Their feedback highlighted that the mobile REDCap platform was not feasible for accurately entering certain portions of the audit form, particularly for the grocery category. Following this pilot of the tool, their feedbacks were incorporated to finalize the tool for the main audits in the web-based REDCap which addressed the issue with grocery section on the audit tool. Prior to audit visits, store hours were verified, and routes were planned using a pre-established schedule. Each auditor carried a printed static map, food audit protocol, an address list, a signed letter of intent from the principal investigator, and an iPad with REDCap for data entry. Since the web-based REDCap was used, auditors connected via their mobile data to complete the audits. Audits lasted approximately 20–60 min depending on the store type. Storefront photographs and GPS coordinates were recorded at each location for verification and geospatial analysis. Data collection commenced in June 2024 and was conducted over six weeks.

### Measures

To assess the consumer nutrition environment in the study site, key consumer nutrition environment dimensions were evaluated using adapted scoring rubric from the previously validated tools [29, 30]. For grocery food store category, the dimensions included the availability, price (affordability), quality, freshness, and variety of food groups. Although data on sweetened beverages and snacks were collected, they were excluded due to inconsistent branding and variety across stores, limiting comparability. For restaurants, healthy specialty, and unhealthy specialty food store categories, availability of healthy food options, the presence of facilitators, and barriers to healthy eating were evaluated. Appendix B and C outline the scoring rubric used to generate values for the relevant dimensions in each food store category. A consumer nutrition environment score (CNES) was computed for each of the 205 stores by summing the scores for availability, quality, affordability, freshness, variety, the availability of healthful options, the presence of facilitators, and barriers to healthy eating as applicable. The CNES were scaled up to 1-100 range to standardize scoring across the varying store types and their dimensions. This was calculated for each food outlet by dividing the actual CNES by the maximum possible score for that store type, then multiplying by 100. This approach allows clearer comparison of the consumer nutrition environments across different store types and categories. Higher CNES means greater or better consumer nutrition environment in terms of available food healthiness.

### Data analyses

Descriptive statistics were used to examine the distribution of the store by types and categories. Mean ± standard deviations (*SD*) were used to describe the standardized CNES by store types and store categories as appropriate. One-way analysis of variance (ANOVA) was conducted to compare the mean CNES across store types and store categories. All statistical tests were with a significance level of *p* < 0.05. All data analyses were conducted using RStudio version 4.3.1.

For community nutrition environment assessment, we examined the spatial distribution of CNES across three store categories (namely healthy specialty, restaurants, and grocery). This spatial analysis was conducted using kernel density analysis (KDA) in ArcGIS Pro version 9. The choice of these three store categories among the four categories studied was based on their relevance to promoting healthier eating options within the community. The default search radius that was derived from the underlying spatial distribution of the data in KDA tool was used for this analysis. This approach reduces subjectivity and reflects the inherent spatial intensity of store locations without imposing an arbitrary scale. Darker colors indicate areas of concentrated supportive food environments.

## Results

### Distribution of the retail food environment by store type and store category

By store type, Table 1 shows a saturation of restaurants, with fast-food and full-service restaurants making up over half (53.7%) of all food outlets. Food trucks including healthy and unhealthy options, comprise about 8.6%. Specialty stores, both unhealthy (14.6%) and healthy (6.8%), also account for a notable share. Food retail outlets that offer food-at-home (FAH) options such as grocery stores and supermarkets were at 4.9% and 4.4% respectively. Convenience stores with gas station made up only 5.4% of all food outlets.

**Table 1:** Distribution of Stores by Type and Category (*N* = 205)

Distribution	*n* (%)
Store Type	
Bar and pub	3 (1.5)
Fast-food restaurant	57 (27.8)
Full-service restaurant	53 (25.9)
Unhealthy specialty store	30(14.6)
Healthy specialty store	14 (6.8)
Healthy food truck	9 (4.4)
Unhealthy food truck	9 (4.4)
Convenience with gas station	11(5.4)
Grocery store	10 (4.9)
Supermarket/supercenter	9 (4.4)
Store Category	
Restaurant	113 (55.1)
Unhealthy specialty store	39 (19.0)
Healthy specialty store	23 (11.2)
Grocery store	30 (14.6)

By store category, the foodscape is heavily skewed, with 55.1% of outlets falling under restaurant category that offer food-away-from-home (FAFH). Unhealthy specialty store category accounted for 19.0% followed by grocery category at 14.6%, while healthy specialty store is least common at 11.2%.

### Characterization of the consumer nutrition environment by store type and store category

Table 2 show the mean standardized CNES by store type and store category. By store types, supermarkets/supercenters and healthy specialty stores had the highest average CNES (µ = 40.9 and µ = 38.6, respectively). Fast-food (µ = 25.0) and full-service restaurants (µ = 20) had moderate scores. On the lower end, unhealthy specialty stores (µ = 13.7), unhealthy food trucks (µ = 11.1), grocery stores (µ = 11.9), and convenience stores with gas stations (µ = 3.6) have low scores. By store category, healthy specialty store and restaurant categories have one of the highest average CNES (µ = 30.2 and µ = 22.3, respectively), followed by grocery category at 17.5.

**Table 2:** Standardized Consumer Nutrition Environment Score (CNES) by Store Type (*N* = 205)

	Mean of Standardized CNES (SD)	Min-Max of Standardized CNES
Store Type		
Fast-food restaurant	25.0ª (13.0)	0-58.8
Full-service restaurant	20ª (8.8)	5.88–41.2
Unhealthy specialty store	13.7ᵇ (14.4)	0–60
Healthy specialty store	38.6ᶜ (14.9)	5–45
Unhealthy food truck	11.1ᵇ (4.2)	5–20
Convenience with gas station	3.6ᵈ (2.1)	1.65–11.3
Grocery store	11.8ᵈ (8.5)	3.02–26.9
Supermarket/supercenter	40.9ᵈ (15.8)	1.92–55.8
Bar and pub	12.7ª (1.7)	11.8–14.7
Healthy food truck	17.2ª (12.8)	5–45
Store Type Category		
Restaurant	22.4ª (11.4)	0-58.8
Unhealthy specialty store	13.1ᵇ (12.8)	0–60
Healthy specialty store	30.2ᶜ (17.4)	5–65
Grocery category	17.5ᵈ (18.6)	1.7–55.8

^ª^Based on maximum Consumer Nutrition Environment score for restaurants. ^ᵇ^Based on maximum Consumer Nutrition Environment score for unhealthy specialty stores. ^ᶜ^Based on maximum Consumer Nutrition Environment score for healthy specialty stores. ^ᵈ^Based on maximum Consumer Nutrition Environment score for grocery stores.

**Fig. 3:**
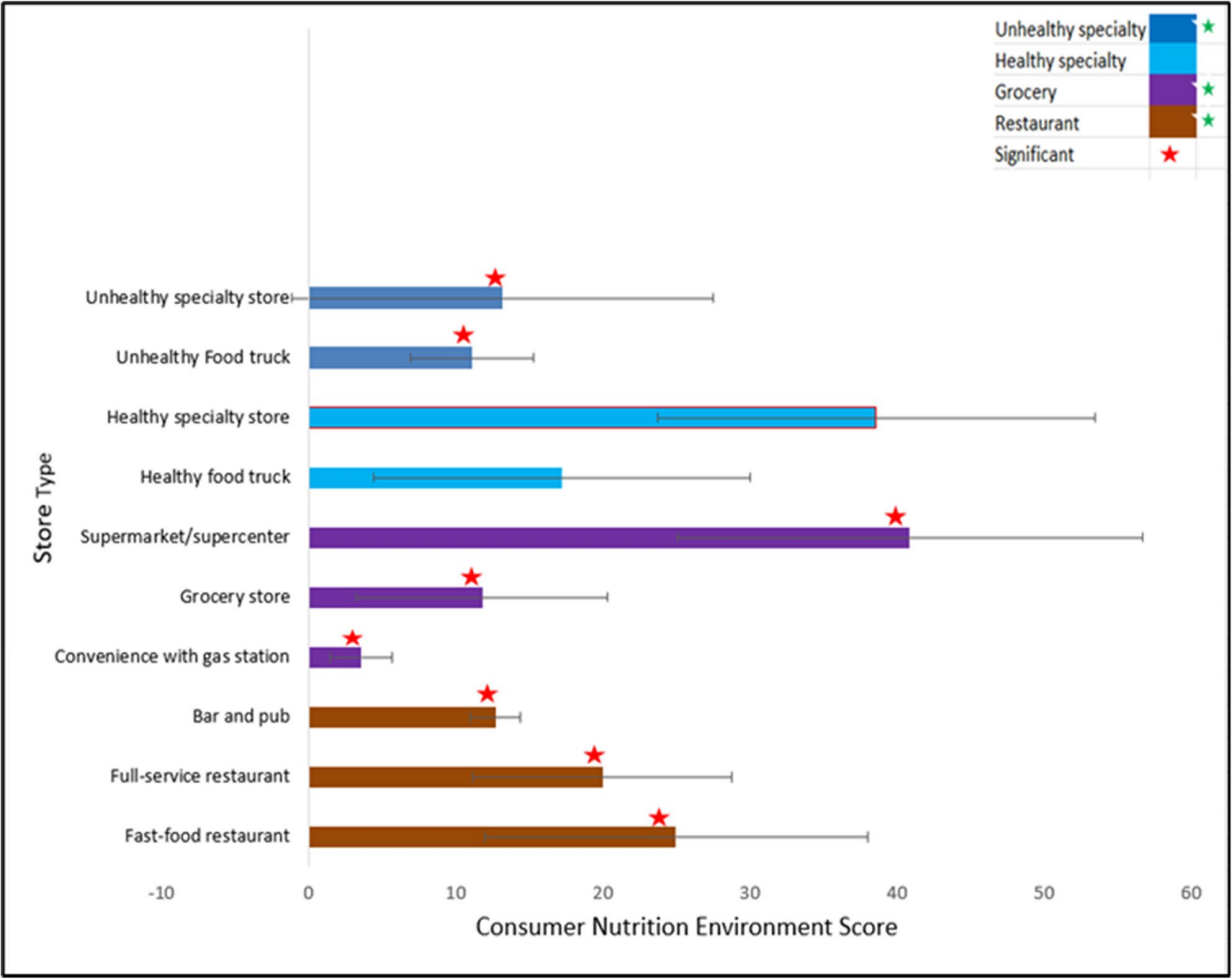
Mean Consumer Nutrition Environment Scores (CNES) across store types with Tukey HSD post-hoc comparisons (*N* = 205). *red star indicates a significant difference for store type and green for store categories

The results of the one-way ANOVA show that standardized CNES differed significantly by store type (F(9, 195) = 13.08, *p* < 0.0001). By store type, Post-hoc Tukey HSD tests in Fig. 3, using healthy specialty store as the reference, showed significantly higher CNES scores for healthy specialty store compared to bar and pub, convenience store with gas station, fast-food restaurant, unhealthy food truck, full-service restaurant, grocery store, and unhealthy specialty store (*p* ≤ 0.03 for all). Unhealthy food truck and unhealthy specialty store had the lowest CNES scores, both significantly lower than healthy specialty store (*p* < 0.001).

By store category, the one-way ANOVA results indicated that at least one store category had a significantly different average CNES compared to the others (*F*(3, 201) = 9.46, *p* < 0.001). Post-hoc Tukey HSD tests show that healthy specialty had significantly higher CNES scores than grocery store (*p* = 0.01). Unhealthy specialty stores had significantly lower CNES scores compared to both healthy specialty (*p* < 0.00001) and restaurant (*p* = 0.001). These differences are also indicated in Fig. 3.

### Characterization of the community nutrition environment by store category

**Fig. 4:**
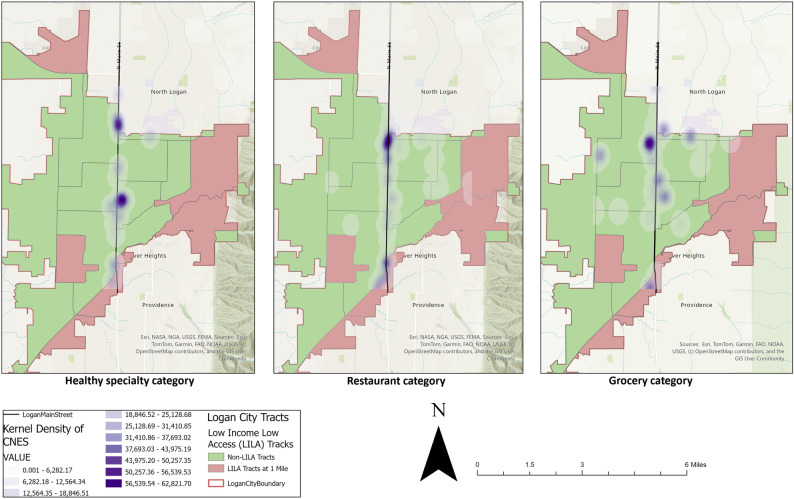
Hotspots of Supportive Nutrition Environment using Consumer Nutrition Environment Scores (CNES) for Healthy Specialty, Restaurant, and Grocery Categories

The spatial distribution of CNES scores in Fig. 4 revealed that healthy specialty stores and restaurants supporting healthy eating were concentrated along the main street corridor, whereas supportive grocery stores were primarily located within the main street food corridor, with only a few scattered pockets in other parts of the city.

## Discussion

This case study provides a comprehensive assessment that integrates availability, quality, price, freshness, and variety dimensions as well as the presence of facilitators, and barriers to healthy eating in a small metropolitan cities retail food environment. Key findings show that Logan’s foodscape is predominantly shaped by fast-food and full-service restaurants at the store-type level and by restaurants at the store-category level, together accounting for more than half of all stores. Beyond this broader trend, our study identified substantial variability in Consumer Nutrition Environment Scores (CNES) across store types and categories. Supermarkets and healthy specialty stores consistently scored highest, offering a better consumer nutrition environment, whereas convenience stores with gas stations demonstrated a poorer environment for healthy choices. At the store-category level, healthy specialty stores have a better consumer nutrition environment.

At the store-category level, healthy specialty category stood out not only in scoring metrics but in their overall alignment with health-promoting retail practices. Comparisons at store type level further demonstrated that healthy specialty stores were significantly different from all other outlets except healthy food trucks, highlighting them as model for a health-supporting food retail environment. To the authors’ knowledge, this is the first study to identify the healthy specialty category as a distinct contributor to a health-supporting food environment. This is an interesting finding, as the healthy specialty stores and food truck offer FAFH options alongside fast-food and full-service restaurants and has potentials to appeal to health-conscious consumers [35]. The dominance of fast food and full-service restaurants in this urban food landscape is not surprising, as Americans now spend over $0.5 trillion more annually on FAFH compared to FAH [36]. Consumers do not select foods based solely on store type or environmental supportiveness. Food choice is a complex decision-making process shaped by individual preferences, taste, cultural norms, time constraints, nutrition knowledge, perceived value, marketing exposure, convenience, and habitual purchasing behaviors [37]. Although the KDA output in Fig. 4 highlights hotspots of supportive restaurant options, these store categories still represent important potential target for improving healthy nutrition access in small metropolitan communities.

Classifying stores by type or category in this study extends beyond descriptive comparisons and highlights specific leverage points for public health and policy interventions. While convenience stores, grocery stores, and supermarkets fall within the broader “grocery” category for their potential to support FAH to certain extent, our findings reported notable differences in the nutritional quality of their offerings. A lower CNES for convenience stores and grocery stores imply that they offer limited availability and variety of fresh, and nutritious foods, making them critical points for improving access to healthier options and reshaping urban food environments to support healthier food choices [18]. The Healthy Food Financing Initiative (HFFI) provide structured support for grocery, corner, and convenience stores in low income, underserved, and rural areas to improve the affordability and accessibility of nutritious food. Expanding program eligibility to include more urban regions could further improve the affordability and accessibility of nutritious food in cities facing food access disparities [38, 39].

The KDA highlights a strong localized density of supportive food outlets in main street corridor, suggesting it functions as a hub for healthier food access. In contrast, the pattern suggests that access to supportive food options is more spatially limited. This spatial analysis approach enabled us to capture not only the geographic distribution of stores but also the extent to which they support healthy eating. However, actual access may depend heavily on transportation networks [40].

This study is not without limitations. While CNES provides a useful proxy for evaluating nutrition environments, it is important to note that the present study does not directly measure dietary behavior or health outcomes. Future research should link CNES with household food purchasing and consumption data to assess whether improvements in food environments translate into meaningful dietary changes. While the kernel density identifies hotspots of supportive nutrition environments the study did not include mobility pattern. Future studies should explore how functionally connected to the population are these hotspots for supportive nutrition environments using transportation network including the existing free transit service offered by CVTD as well as incorporate qualitative data (e.g., residents’ and storeowners’ perspectives), which could enrich understanding of facilitators and barriers to healthy food retailing and access. Finally, examining whether census tracts with lower access to supportive food environments differ in racial, ethnic, or socioeconomic composition may help clarify potential spatial inequities in local food access. The strength of the study lies in the composite index approach used, which quantified nutrition environments and identified store types and categories that may be targeted to improve the healthfulness of retail offerings.

## Conclusion

This study provides a nuanced understanding of the consumer nutrition environment across different retail formats in Logan, Utah. Supermarkets and healthy specialty stores emerged as supportive of nutritious food choices. The dominance of fast-food and full-service restaurants, though may be seen as a challenge, presents an opportunity to engage these outlets in promoting healthier options, creating partnerships, or implementing interventions that turn their prevalence into a positive contribution to the urban food environment in a small metropolitan area. The findings on grocery category highlight the potential for the Healthy Food Financing Initiative (HFFI) to enhance access to nutritious foods low-income low access urban communities.

The spatial analysis suggests Logan’s main street corridor as a hub for healthier food access, though access in outlying neighborhoods remains limited. This concentration may not only support small businesses but also fosters social interaction, contributing to a vibrant local economy and community life.

Importantly, this research highlights that improving retail food environments is not solely a matter of increasing physical access but also involves enhancing the nutritional quality of available options and supporting informed food choices. Moving forward, public health efforts should prioritize policy alignment, ongoing retail environment monitoring, and collaborative partnerships with local retailers to advance equitable access to healthy foods. By integrating these strategies, communities can foster more health-supportive foodscapes that promote dietary quality and reduce nutrition-related health disparities.

## Supplementary Information

Supplementary Material 1.

## Data Availability

The dataset supporting the conclusions of this article will be made available upon request.
