# Supplementary material for: Retail food environment assessments: a case study of consumer and community nutrition environment perspectives of Logan, Utah
Source: BMC Public Health. 2026 May 16;26:2111. doi: 10.1186/s12889-026-27398-x (PMC13352832; doi:10.1186/s12889-026-27398-x)
Supplement: Supplementary file 1 — Supplementary Material 1. [file 12889_2026_27398_MOESM1_ESM.docx]

**Appendix A: Food Store Types, Categories, and Definitions**

| Food Store Type | Definition | Store Category |
| --- | --- | --- |
| Grocery stores | Stores that primarily sell a variety of fresh and prepared food products, sell other items too but have a larger portion of their aisles dedicated to food compared to other items. This includes small grocery stores, ethnic grocery stores, and corner stores | Grocery category includes all stores that sell a general range of food products, fresh or packaged that support home meal planning to some extent |
| Supermarkets/supercenters | Supermarkets are large store that sells a wide variety of food and household products, usually organized into different sections. Supercenters are larger than supermarkets and sell at discounted prices. They also sell clothing and home goods but have a section or complete block for groceries. |  |
| Convenience stores | Stores located at a place with convenient transportation that primarily sells convenience goods and food products that are already prepared and packaged. E.g. convenience stores (with or without gas stations). |  |
| Fast-food outlets | Eating places that sell pre-prepared or quickly prepared food at a counter that is likely highly processed. | Restaurants are establishments that provide food services where patrons typically order or select items and pay before eating on-site or taking the food to go. |
| Full-service restaurants/ sit-down restaurants | Eating places where patrons typically order from a waiter, can be seated for dine-in and pay after eating. |  |
| Bars and pubs | Drinking place/relaxation center where a bar attendant serves alcoholic and non-alcoholic beverages. |  |
| Specialty stores | Pre-prepared or quickly prepared food at a counter that may be healthy or unhealthy. | Healthy and unhealthy specialty stores offer curated menus with distinctive identities. Healthy specialty stores typically provide nutritious options such as smoothies, tacos, and sandwiches, while unhealthy specialty stores focus on items like slushes and ice cream. |
| Food trucks | Large, motorized vehicle that offers a variety of food options, from frozen or prepackaged items to meals freshly prepared on-site. |  |

^* Healthy and unhealthy specialty stores classification was informed by the Dietary Guidelines for Americans^

**Appendix B: Scoring for the Different Dimensions of the Consumer Nutrition Environment for Grocery Store Category**

|  | Food Groups | | | | | |  |
| --- | --- | --- | --- | --- | --- | --- | --- |
| Measures | **Grains**  **Score** | **Fruits**  **Score** | **Vegetables**  **Score** | **Meat**  **Score** | **Dairy and Eggs Score** | **Legumes and Nuts Score** | **Maximum Subscore for Consumer** **Nutrition Environment Scoreª** |
| Availability | 11 | 6 | 9 | 11 | 5 | 10 | 52 |
| Quality | N/A | Good = 2, Fair =1, Bad =0 | | | N/A | N/A | 52 |
| Freshness# | N/A | 25-50% =1, 51-75% =2, 75%+ =3 | | | N/A | N/A | 78 |
| Variety | N/A | 1-4 =1, 5-9 =2, 10+ =3 | | | N/A | N/A | 78 |
| Affordability (Price)* | (≤Q1 = 2), (>Q1 to <Q3) = 1, (≥Q3) = 0 | | | | | | 104 |

^*Pricing was standardized per pound or unit and recoded based on quartile distributions. #Items meeting 25–50% of optimal quality standards = 1 point, 51–75% = 2 points, and >75% = 3 points. ªMaximum Consumer Nutrition Environment score was 364 based on the summation of the possible subscore for each measure. N/A implies that quality, freshness, and variety do not apply to Grain, “Dairy and Eggs” and “Legumes and Nuts” Scores.^

**Appendix C: Scoring for the Different Dimensions of the Consumer Nutrition Environment Restaurants and Specialty Store Categories**

| Measures | Restaurants  Score | Healthy Specialty Store  Score | Unhealthy Specialty Store  Score |
| --- | --- | --- | --- |
| Availability of Healthy Options | 8 | 5 | 5 |
| Facilitators of Healthy Eating | 10 | 11 | 10 |
| Barriers to Healthy Eating | 5 | 4 | 5 |
| Kid’s Menu | 11 | N/A | N/A |
| Maximum Consumer Nutrition Environment Scoreª | 34 | 20 | 20 |

^N/A implies that Kid’s Menu does not apply to Healthy and Unhealthy Specialty Store Scores. ªMaximum Consumer Nutrition Environment score was based on the summation of the possible subscore for each measure.^
